# Who considers alternative employment pathways among International Medical Graduates in Canada?

**DOI:** 10.1371/journal.pone.0355462

**Published:** 2026-08-13

**Authors:** Tanvir C. Turin, Nashit Chowdhury, Deidre Lake, Mohammad Z. I. Chowdhury

**Affiliations:** 1 Department of Family Medicine, Cumming School of Medicine, University of Calgary, Calgary, Alberta, Canada; 2 Department of Community Health Sciences, Cumming School of Medicine, University of Calgary, Calgary, Alberta, Canada; 3 Alberta International Medical Graduates Association, Calgary, Alberta, Canada; 4 Provincial Research Data Services, Health Shared Services, Calgary, Alberta, Canada; 5 Department of Psychiatry, Cumming School of Medicine, University of Calgary, Calgary, Alberta, Canada; University of Washington, UNITED STATES OF AMERICA

## Abstract

This study aims to examine which International Medical Graduates (IMGs) consider alternative employment pathways in Canada. IMGs are physicians whose country of practice differs from the country where they obtained their primary medical qualification. Due to the limitations and uncertainties of this career pathway, many IMGs opt for alternative career pathways (ACPs), which include non-physician roles that enable IMGs to apply their transferable knowledge, skills, and experience. This study aimed to analyze which IMGs choose alternative careers for job market integration in Canada. We carried out an anonymous, open online survey of IMGs living in Canada between November 2019 and June 2020. We recruited through IMGs-serving organizations, professional networks, social media platforms, and IMG champions. The survey collected information on sociodemographic characteristics, professional background, and consideration of ACPs. Descriptive analyses and multivariable logistic regression were used to examine factors associated with ACP consideration. Of the 1,740 survey responses received, 1,210 respondents with complete information on the primary outcome were included in the analysis. Thus, the final sample used for this analysis included 1,210 responses from IMGs who were not currently working in clinical settings as partially/fully licensed physicians and who provided responses to our key question about considering ACPs. This sample included both those considering ACPs (683 respondents, 56.45%) and those not considering ACPs (527 respondents, 43.55%), enabling comparison between these two groups of IMGs in Canada. Among participants aged ≥ 50 years, the majority were considering ACPs (75.65%). Compared to migrants who had been in Canada for <3 years, participants with longer stays in Canada (>15 years) were less likely to consider ACP, OR: 0.82, 95% CI: 0.47–1.43. An opposite trend in ACP consideration was observed between graduates within <3 years and earlier graduates (>15 years), OR: 3.41, 95% CI: 1.39–8.38. Despite persistent barriers and low residency match (13%−18%) in Canada, more than half of respondents reported considering alternative career pathways. IMGs aged 50 or older and those with longer time since graduation were more likely to consider alternative careers, whereas gender and length of stay in Canada were not significantly associated with ACP consideration. These insights from this study can guide targeted future support and career strategies to better facilitate IMGs’ professional integration into the Canadian workforce, supporting their transition to fulfilling and meaningful employment.

## Introduction

Canada has been at the forefront of the global trend of developing human capital-oriented immigration and implemented a point-based high-skilled immigration system designed to attract highly qualified professionals, including International Medical Graduates (IMGs) [1–3]. International medical graduates (IMGs) are physicians whose country of practice differs from the country where they obtained their primary medical qualification (PMQ) [4,5]. In the Canadian context, IMGs are individuals who obtained their PMQ outside Canada and seek professional integration into the Canadian healthcare system. IMGs include both permanent residents and citizens – whether by birth or naturalization – including those who immigrated to Canada. IMGs form a substantial component of physician workforces across many developed countries, including the United States (US), the United Kingdom (UK), Australia, Germany, France, etc. Approximately 25% of practicing physicians in the United States are IMGs in 2023 [6], while the UK reported around 138,405 IMGs in practice in 2024, representing 42% of the licensed physician workforce [7]. According to OECD Health at a Glance 2023, foreign-trained physicians accounted for approximately 19% of physicians across Organization of Economic Cooperation and Development (OECD) countries in 2021, with corresponding shares of approximately 31% in Australia, 14% in Germany, and 12.3% in France [8]. These figures underscore the dependence of developed countries on IMGs and highlight the importance of understanding their professional integration in Canada [9]. In 2023, IMGs represented 27% of Canada’s 97,384 physicians [10], while an estimated 259,695 internationally educated healthcare professionals immigrated to Canada by 2021, of whom 15.2 percent were physicians [11]. About half of the IMGs immigrated between the ages of 25 and 34 years, making them a substantial component of Canada’s skilled migrant population [12]. Yet, despite Canada’s persistent physician shortage, many IMGs encounter substantive barriers to becoming a licensed practicing physician in Canada [9,13–15]. Canada’s healthcare system is facing a severe physician shortage. Canada ranks 26^th^ among the 34 OECD countries and faces shortage of 22823 family physicians while producing only 1300 new medical graduates annually [14]. Although Canada recruits highly skilled IMGs partly to address physician shortages, the pathway to physician licensure remains highly restrictive. IMGs generally must complete credential verification, licensing examinations, and residency training through the Canadian Resident Matching Service (CaRMS) system, which remains the principal route to physician practice [15,16]. However, residency opportunities for IMGs are limited. While Canadian and US medical graduates tend to maintain residency match rates over 90% [5], the typical match rate for IMGs has ranged from 13% to 18% [16]. As a result, many IMGs spend years navigating licensing requirements while facing prolonged professional and financial uncertainty.

The low success rate in residency matching – the primary route to physician licensure in Canada, creates a major bottleneck for IMGs and often redirects them toward alternative career pathways (ACPs) [15,16]. ACPs include non-physician roles in healthcare and other areas that enable IMGs to apply their transferrable knowledge, skills, and experience. Some IMGs pursue ACPs as permanent career transitions, whereas others take them as a temporary strategy while continuing efforts to return to their original profession despite significant financial and personal investment, with only a handful ultimately succeeding [17–19]. Existing evidence suggests that Canadian immigration policies successfully attract highly skilled healthcare professionals but remain insufficiently coordinated with the licensing and the health workforce system, creating barriers to professional integration [11]. Consequently, many IMGs experience uncertainty regarding whether to continue pursuing medicine or transition into alternative careers. Although demographic information on IMGs practicing as physicians in Canada is tracked by the Canadian Institute for Health Information (CIHI) [20], information on IMGs pursuing alternative careers remains largely unavailable. Similarly, little is known about which IMGs are more likely to consider ACPs or how demographic differences shape such decisions.

Understanding under what conditions IMGs consider ACPs is important not only for Canada but also for countries increasingly dependent on internationally trained physicians to address healthcare workforce shortages. Many high-income countries rely heavily on IMGs while maintaining complex licensing systems that may delay professional integration. Better understanding the characteristics associated with ACP consideration may therefore help policymakers and support organizations design targeted supports that mitigate professional deskilling and improve workforce utilization. This study, therefore, attempts to examine the demographic characteristics of IMGs who intend to pursue alternative careers and those who do not. This research complements our earlier work [21], which investigated employment patterns among IMGs already working in various alternative career roles, by focusing specifically on IMGs who consider alternative careers regardless of their current employment role or status. However, the sample and objective of this study differ by focusing specifically on those who consider alternative careers as a potential or planned career option instead of limiting their focus to only physician roles, regardless of their current employment role and status. This need emerged through our collaboration with IMG-serving organizations such as the Alberta International Medical Graduates Association (AIMGA), which actively supports IMGs in their pursuit of alternative careers. In doing so, we co-created this study by incorporating the lived experience of the IMG researchers of this study (TCT and NC), and AIMGA (DL).

## Methods

### Public involvement

This study is a component of our community-engaged program of research led by the research team in collaboration with IMG community members and the Alberta International Medical Graduates Association (AIMGA), focused on the professional integration of IMGs in Canada and related system factors [2,22]. The research team includes IMG community members (TCT and NC) with lived experience of alternative career pathways in Canada, who contributed to study design, interpretation of findings, and knowledge co-creation. AIMGA (DL), an organization supporting all different types of IMGs, including those pursuing careers in medicine and those considering ACPs, collaborated in identifying the research need, study design, survey development, participant recruitment, and interpretation of findings. Study findings and dissemination activities were conducted collaboratively with community partners to facilitate feedback, learning, and capacity-building [2,22].

### Survey population

The survey targeted all IMGs living in Canada who had completed medical training (PMQ) outside Canada and were not currently working in clinical settings as partially or fully licensed physicians in Canada.

### Survey questionnaire

The voluntary, anonymous, and open online survey comprising questions on participant demographics and if they are in ACPs. Here, the “open survey” indicates a survey open for each visitor of the site. The survey was developed by drawing upon the current literature, as well as the collective expertise of our team on this topic gained through collaborative research and IMG community involvement over the past number of years [17–19]. For this study, we examined IMGs’ intentions to consider ACPs by using the survey question: *‘Are you considering for an alternative career pathway?’* (Question 29, see S1 Appendix). Respondents answering *‘Yes’* were classified as considering ACPs. Other relevant questions in the survey were closed-ended with a couple of open-ended questions explaining ‘other’ options, if applicable.

We categorized length of stay in Canada into five groups: less than 3 years, 3–5 years, 6–10 years, 11–15 years, and more than 15 years. While the categories generally follow five-year intervals, we split the first interval to distinguish those who have reached the three-year mark required for Canadian citizenship following permanent resident status – significant milestone for IMGs. To maintain consistency in category structure, we applied a similar rationale to the variable measuring years since graduation, ensuring both variables include five comparable intervals. In the questionnaire, age was the only variable for which respondents selected their responses from predefined categories (29 years or younger, 30–39 years, 40–49 years, and 50 years or over). The median and the mean number of questions per page were 3 and 3.3, respectively, spread across 13 pages. Skip logic was used in the survey platform (Qualtrics survey software) [23] to allow participants to skip questions that did not apply to them.

The survey did not collect any personal information and was pilot-tested a priori by IMG members involved with the research team. Our survey platform provided the option to prevent duplicate submissions from a single Internet Protocol (IP) address. However, no IP address-related information was retrieved from the survey software as a data point. Additionally, a $10 gift card was offered to 50 randomly selected participants who completed the survey. The contacts for those willing to contest the raffle draw were asked using a separate form linked at the end of the survey, thus conserving the privacy and confidentiality of the survey responses.

### Informed consent

Implied consent was sought in this survey by adding the study information to the landing page of the survey. So, participants provided digital consent by proceeding with the survey.

### Ethical approval

The study received approval from the University of Calgary’s Conjoint Health Research Ethics Board (REB19-1198) and meets the ethical standards laid out in Canada’s Tri-Council Policy Statement: Ethical Conduct for Research Involving Humans (TCPS 2). The survey had a landing page describing the survey information and consent was implied when participants clicked the ‘Go to Survey’ button from this page.

### Survey dissemination

A convenience sampling method [24] was employed in which various organizations in Canada providing support to IMGs in their career resettlement were approached and requested to circulate the anonymous online survey among their members via email/other communication systems. To facilitate this, we provided the study information in the form of a poster and an email body with the survey link. Further, the study was regularly advertised on various social media platforms such as Facebook and LinkedIn. There were multiple repeat postings on social media platforms to maximize the participation of IMGs in the survey. We surveyed between November 2019 and June 2020.

### Data analysis

All the variables used in this study were categorical. Accordingly, we first conduct descriptive analyses to summarize participant characteristics and compare IMGs who considered alternative career pathways (ACPs) with those who did not. We used χ2 tests or Fisher’s exact tests for the comparison, as they met the respective assumptions [25,26]. To examine factors associated with ACP consideration, we also estimated multivariable logistic regression models [27,28] to examine the relationship between the ACP group and non-ACP groups. We selected the variables based on theoretical relevance and prior literature on professional integration of IMGs. We first estimated unadjusted models and then adjusted for relevant sociodemographic characteristics like age, gender, current living location, country of origin, country of medical graduation, i.e., PMQ, length of stay in Canada, years passed after graduation, and the highest level of practice before coming to Canada. Odds ratios (ORs) with 95% confidence intervals (CIs) were reported. Statistical significance was assessed at p < 0.05. Given the theory-informed nature of the regression analyses and the limited number of prespecified comparisons, formal adjustment for multiple comparisons was not applied. Findings were interpreted considering effect sizes, confidence intervals, and overall consistency. We carried out all statistical analysis using STATA version 15.1 (StataCorp, College Station, Texas, USA). S2 Appendix contains the checklist for reporting the results of Internet e-surveys (CHERRIES) [29].

## Results

We collected a total of 1,740 responses. On average, participants completed 85.92% of the questionnaire, with 1,149 (76.75%) participants completing the survey in its entirety. Incomplete questionnaires with missing responses were also included in the analysis whenever we found valid response to the primary question of our interest regarding alternative career consideration. For our empirical analysis, we have included 1,210 respondents who indicated if they are considering alternative careers or not, regardless of their current employment status or field, as the analysis is based on their intention, not their present employment. Respondents with incomplete questionnaires, including missing responses to the primary question of interest regarding alternative career consideration, were excluded from the analysis. Consequently, we excluded 30.46% of total respondents, yielding a final sample of 1210 responses. Among the respondents, 527 (43.55%) were not considering any ACPs, whereas 683 (56.45%) were considering one.

Table 1 shows the characteristics of the IMGs across the groups who were considering ACPs and those who were not. With the exception of gender and country of origin, the demographic characteristics of the participants differed significantly (p < 0.05) between those who were considering taking an ACP and those who were not. Among participants of age ≥ 50 years, the majority were considering ACPs (75.65%). The proportion of participants considering ACPs was higher among the age group of 40–49 years (61.46%) and 30–39 years (56.59%) as well. South Asia is the most common region of origin among respondents, while Europe and Central Asia are the least represented. The respondents included in this study are from ten provinces. The majority reside in Ontario, whereas the fewest are from Prince Edward Island. No respondents were recorded from any of the territories.

**Table 1:** Characteristics of the survey respondent IMGs by choosing alternative career pathways (ACP) or not.

Variables	Considering ACP		
	No, *n* (%)	Yes, *n* (%)	*p*-value^#^
*N*	527 (43.55)	683 (56.45)	
*Age group (years)*			
≤ 29	138 (59.48)	94 (40.52)	< 0.001
30–39	237 (43.41)	309 (56.59)	
40–49	121 (38.54)	193 (61.46)	
≥ 50	28 (24.35)	87 (75.65)	
*Gender*			
Female	373 (45.43)	448 (54.57)	0.082
Male	148 (39.15)	230 (60.85)	
Other	1 (33.33)	2 (66.67)	
*Province (currently living)*			
Alberta	169 (42.04)	233 (57.96)	< 0.001
British Columbia	47 (43.93)	60 (56.07)	
Manitoba	20 (76.92)	6 (23.08)	
New Brunswick	4 (66.67)	2 (33.33)	
Newfoundland and Labrador	2 (6.90)	27 (93.10)	
Nova Scotia	6 (37.50)	10 (62.50)	
Nunavut	0 (0.00)	0 (0.00)	
Ontario	220 (44.09)	279 (55.91)	
Prince Edward Island	0 (0.00)	1 (100.00)	
Quebec	28 (45.16)	34 (54.84)	
Saskatchewan	13 (50.00)	13 (50.00)	
*Country/region of origin*			
East Asia and Pacific	15 (30.61)	34 (69.39)	0.117
Europe and Central Asia	20 (42.55)	27 (57.45)	
North America, Latin America, and the Caribbean	49 (43.36)	64 (56.64)	
Middle East and North Africa	137 (45.36)	165 (54.64)	
South Asia	208 (42.98)	276 (57.02)	
Sub-Saharan Africa	92 (52.27)	84 (47.73)	
*Country/region where medical graduation was obtained*			
East Asia and Pacific	20 (31.75)	43 (68.25)	0.034
Europe and Central Asia	50 (51.55)	47 (48.45)	
North America, Latin America, and the Caribbean	41 (38.32)	66 (61.68)	
Middle East and North Africa	129 (43.88)	165 (56.12)	
South Asia	195 (43.72)	251 (56.28)	
Sub-Saharan Africa	83 (52.53)	75 (47.47)	
*Length of stay in Canada (years)*			
< 3	179 (48.64)	189 (51.36)	0.004
3–5	128 (46.04)	150 (53.96)	
6–10	112 (35.44)	204 (64.56)	
11–15	39 (43.82)	50 (56.18)	
> 15	44 (36.07)	78 (63.93)	
*Years passed after graduation*			
< 3	69 (71.88)	27 (28.13)	< 0.001
3–5	77 (50.33)	76 (49.67)	
6–10	131 (45.80)	155 (54.20)	
11–15	107 (46.12)	125 (53.88)	
> 15	134 (31.38)	293 (68.62)	
*Highest level of practice before coming to Canada*			
Medical student	98 (55.37)	79 (44.63)	0.001
Resident	90 (48.13)	97 (51.87)	
Attending physician/ hospital physician	67 (42.41)	91 (57.59)	
Consultant physician/ specialist	99 (35.61)	179 (64.39)	
General practitioner/ family physician	165 (43.08)	218 (56.92)	
Department head	5 (25.00)	15 (75.00)	

Note: Due to missing responses, some variable cell counts may not add up to total population number. ^#^p-value from Pearson Chi-square test or Fisher’s exact test as appropriate. Question 29 of the survey asks whether respondents are considering an ACP. Those who respond affirmatively receive follow-up questions to collect detailed information about their specific ACP.

Table 2 shows the multivariable-adjusted odds ratio (OR) and 95% confidence interval (CI) associated with the likelihood of choosing ACPs. Age is positively associated with choosing an ACP, with those 50 years and older having a 2.43 times higher likelihood of choosing ACPs compared to the reference category, ≤ 29 age group (OR:2.43, 95% CI:1.02–5.80). We observed a dose-response relationship between the number of increased years passed after graduation and the likelihood of choosing ACP. In this multivariable-adjusted analysis, gender was not a significant factor among IMGs in terms of choosing ACP (Female OR:0.93, 95% CI: 0.69–1.25). The length of stay in Canada also did not have a significant association with ACP consideration. The OR was 0.86 (95% CI:0.60–1.22) for individuals who stayed in Canada for 3–5 years, 1.10 (95% CI:0.75–1.61) for 6–10 years, 0.70 (95% CI:0.40–1.25) for 11–15 years, and 0.82 (95% CI:0.47–1.43) for individuals who stayed in Canada for more than 15 years, when compared to the reference group (< 3 years). Compared to IMGs residing in Alberta, those residing in Manitoba were significantly less likely (OR: 0.23, 95% CI: 0.09–0.60) to opt for alternative careers. IMGs residing in Newfoundland and Labrador were more likely to opt for alternative careers, (OR: 1.21, 95% CI: 0.10–14.59). However, due to the wide confidence interval, these results should be interpreted with caution. Though statistically insignificant, IMGs from South Asia (OR: 1.29, 95% CI: 0.42–4.02) and Europe and Central Asia (OR: 1.21, 95% CI: 0.31–4.81) were more likely to opt for alternative careers in comparison to those from East Asia and Pacific region. On the other hand, IMGs from Middle East and North Africa (OR: 0.37, 95% CI: 0.09–1.54) and Sub-Saharan Africa (OR: 0.51, 95% CI: 0.12–2.18) were less likely to opt for alternative careers.

**Table 2:** Factors associated with choosing ACP among the survey respondent IMGs.

Variables	Multivariable adjusted OR^#^ (95% CI†)	p-value
*Age group (years)*		
≤ 29	Reference	
30–39	1.19 [0.71–2.00]	0.502
40–49	1.31 [0.65–2.65]	0.453
≥ 50	2.43* [1.02–5.80]	0.046
*Gender*		
Female	0.93 [0.69–1.25]	0.644
Male	Reference	
Other	1	
*Province (currently living)*		
Alberta	Reference	
British Columbia	0.95 [0.59–1.54]	0.849
Manitoba	0.23** [0.09–0.60]	0.003
New Brunswick	0.25 [0.03–2.30]	0.220
Newfoundland and Labrador	1.21 [0.10–14.59]	0.883
Nova Scotia	0.95 [0.32–2.81]	0.925
Nunavut		
Ontario	0.90 [0.67–1.22]	0.509
Prince Edward Island	1	
Quebec	0.90 [0.49–1.68]	0.751
Saskatchewan	0.58 [0.25–1.33]	0.197
*Country/region of origin*		
East Asia and Pacific	Reference	
Europe and Central Asia	1.21 [0.31–4.81]	0.785
North America, Latin America, and the Caribbean	0.83 [0.24–2.90]	0.764
Middle East and North Africa	0.37 [0.09–1.54]	0.172
South Asia	1.29 [0.42–4.02]	0.658
Sub-Saharan Africa	0.51 [0.12–2.18]	0.365
*Country/region where medical graduation was obtained*		
East Asia and Pacific	Reference	
Europe and Central Asia	0.60 [0.19–1.87]	0.376
North America, Latin America, and the Caribbean	1.52 [0.48–4.86]	0.477
Middle East and North Africa	1.36 [0.36–5.14]	0.647
South Asia	0.57 [0.21–1.57]	0.276
Sub-Saharan Africa	0.90 [0.23–3.58]	0.882
*Length of stay in Canada (years)*		
< 3	Reference	
3–5	0.86 [0.60–1.22]	0.383
6–10	1.10 [0.75–1.61]	0.619
11–15	0.70 [0.40–1.25]	0.227
> 15	0.82 [0.47–1.43]	0.484
*Years passed after graduation*		
< 3	Reference	
3–5	2.25* [1.20–4.23]	0.012
6–10	2.66* [1.26–5.60]	0.010
11–15	2.62* [1.18–5.83]	0.018
> 15	3.41** [1.39–8.38]	0.008
*Highest level of practice before coming to Canada*		
Medical student	Reference	
Resident	0.84 [0.50–1.42]	0.517
Attending physician/ hospital physician	0.92 [0.53–1.60]	0.762
Consultant physician/ specialist	0.88 [0.49–1.57]	0.664
General practitioner/ family physician	0.87 [0.53–1.43]	0.584
Department head	1.41 [0.43–4.60]	0.567

Note: ^#^ OR = odds ratio; † CI = confidence interval. Statistically significant comparisons are indicated by symbols: * p < 0.05, ** p < 0.01, *** p < 0.001. Question 29 of the survey asks whether respondents consider pursuing an alternative career pathway. If they respond affirmatively, the survey follows up with questions to gather detailed information about their specific ACP.

Response options related to the ‘age’ variable only were combined in survey questionnaire.

## Discussion

More than half of the respondents (56.45%) reported considering ACPs, while 43.55% were not considering alternative careers. In the multivariate analysis, IMGs aged 50 years and above, and those with longer time since graduation, were more likely to consider ACPs. Specifically, respondents aged 50 years or older had higher odds of considering ACPs than younger respondents, while respondents who had graduated longer ago consistently showed higher odds of considering alternative careers. Length of stay in Canada was not significantly associated with ACP consideration after multivariate adjustment. We observed provincial variation, but few provincial-level differences emerged as statistically significant. These findings suggest that demographic and professional characteristics may shape how IMGs navigate career uncertainty and professional integration in Canada.

This study has several strengths. To our knowledge, it is one of the first community-engaged national surveys examining factors associated with considering ACPs among IMGs in Canada. The study was co-developed in collaboration with knowledge users and community partners, including IMG community members and AIMGA, which helped ensure the relevance and appropriateness of the questionnaire for the intended population and facilitated implementation of the generated knowledge [22,30–33]. Further, recruitment through multiple channels across Canada enabled Participation from IMGs residing in different provinces and originating from diverse geographical regions.

Nevertheless, several limitations should be considered when interpreting the findings. Due to the absence of a centralized contact list for IMGs pursuing either physician or alternative career pathways, we employed an open-access convenience sampling approach. Recruitment occurred through multiple IMG-serving organizations, professional networks, and social media platforms; therefore, the total number of IMGs reached through recruitment efforts could not be determined, preventing estimation of a response rate. Although recruitment occurred primarily through trusted IMG-serving organizations and networks, independent verification of IMG status was not feasible because the survey was anonymous. Similarly, automated or bot responses cannot be completely ruled out, although targeted recruitment and IP-based duplicate prevention likely reduced this risk. Further, because the study used a cross-sectional design and a non-probability sample, findings cannot be interpreted causally or generalized to the broader IMG population in Canada. Online surveys may also be affected by reporting inaccuracies, incomplete responses, or variation in respondent engagement. In addition, the survey period (November 2019 to June 2020) overlapped with the onset of the COVID-19 pandemic, which may have influenced the overall number of responses obtained and the rate of survey non-completion. As with any voluntary online survey, the possibility of self-selection bias cannot be excluded, as IMGs with greater interest in alternative career pathways may have been more inclined to participate. Although a precise sampling denominator was unavailable, AIMGA, the largest participating IMG-serving organization, had ~ 2,000 actively served members at the time of the survey, while comparable figures for the other recruitment channels could not be obtained.

Our findings align with prior research highlighting persistent barriers to professional integration among IMGs in Canada. Wang et al. reported that many IMGs experience substantial challenges when pursuing alternative careers and that only 53% of respondents in their study were employed in ACPs [13]. Similar to our findings, their study highlighted ongoing uncertainty surrounding career transitions among IMGs navigating barriers to physician licensure. However, important methodological differences distinguish the present study from prior work. While Wang et al. primarily examined IMGs already employed in ACPs, our study focused on factors associated with considering ACPs regardless of respondents’ current employment status. This distinction is important because consideration of ACPs represents an earlier stage in career decision-making and may better capture uncertainty surrounding professional integration.

Our findings also complement previous Canadian evidence suggesting that IMGs may experience limited access to alternative career services and continuing education opportunities relevant to ACPs [13,34]. Wang et al. further reported that the educational and training needs for ACPs are distinct from those associated with physician residency pathways [13] and that many IMGs relied primarily on online job searches or informal social networks to identify alternative opportunities. Together, these findings suggest that career transition support for IMGs remains fragmented and may not adequately address the distinct challenges associated with ACP pursuit. However, unlike prior studies focused primarily on employment outcomes, the present study provides additional insight into demographic and professional characteristics associated with pursuing ACPs. Ultimately, a well-implemented outreach strategy can help IMGs achieve greater career stability and success and contribute to a stronger and more diverse health workforce [13,19,35].

The findings suggest that ACP consideration among IMGs may be shaped by professional timing and career trajectory. Respondents aged 50 years and above and those with longer time since graduation were more likely to consider ACPs, which may reflect prolonged barriers to physician licensure, changing career expectations, financial pressures, or reassessment of career feasibility over time. While this interpretation should be approached cautiously, it may indicate that continued unsuccessful attempts to secure physician licensure influence decisions to pursue non-physician careers.

The findings also highlight potential opportunities for strengthening support systems for IMGs. Since ACPs may represent either permanent career transitions or temporary pathways during continued attempts to enter medicine, targeted supports – including career counselling, bridging opportunities, networking, and information regarding non-physician healthcare careers – may help IMGs navigate career uncertainty more effectively. Improving awareness of alternative professional pathways may also help reduce professional deskilling and underutilization of immigrant physician talent. Given ongoing physician shortages and increasing reliance on internationally trained physicians across many high-income countries, including Canada, more effective integration pathways may contribute to better workforce utilization while reducing economic and professional uncertainty for IMGs. At the same time, interpretation of province-level differences should remain cautious because substantial heterogeneity exists within provinces regarding labor markets, support services, and healthcare systems.

Several important questions remain unanswered in our study. Future research using longitudinal designs may help clarify how ACP consideration evolves over time and whether professional trajectories change as IMGs spend more years attempting physician licensure. Public records indicate that IMGs represented 27% of Canada’s physician workforce in 2023 [10] and that internationally educated healthcare professionals constitute a substantial component of skilled migration to Canada. [11] However, existing administrative data provide limited insight into professional trajectories following migration. Future research integrating migration, licensure, and workforce participation records may improve understanding of how migration pathways shape professional integration and ACP decisions among IMGs.

Qualitative and mixed-methods research may deepen understanding of how IMGs navigate uncertainty, interpret barriers to physician licensure, and make decisions regarding ACPs. In particular, qualitative work could provide important insight into how financial pressures, family circumstances, professional identity, institutional barriers, and social networks influence career transitions among IMGs. Such evidence may help policymakers, educators, and support organizations design more responsive and equitable professional integration strategies.

**Key Messages**

**What is already known about this subject?**
  - International Medical Graduates (IMGs) frequently encounter substantial barriers to physician licensure in Canada, leading them to pursue alternative careers.
  - Despite being highly skilled healthcare professionals, IMGs often face regulatory, credentialing, and residency-related barriers that delay professional integration and may redirect them toward non-physician career options.
**What are the new findings?**
  - More than half of IMGs in this study (56.45%) reported considering ACPs.
  - IMGs aged 50 years or older and those with a longer time since graduation were more likely to consider alternative careers.
  - Length of stay in Canada and gender were not significantly associated with ACP consideration after adjustment for sociodemographic and professional characteristics.
**How might these results change the focus of research or clinical practice?**
  - These findings underscore the necessity for targeted supports, including career counseling, networking, and information on non-physician healthcare roles, to better support IMGs considering ACPs.
  - Future research using longitudinal, qualitative, and administrative data approaches is needed to better understand how IMGs navigate career uncertainty and professional integration over time.

## Supporting information

S1 File S1 Appendix Questionnaire. (DOCX)

S2 File S2 Appendix CHERRIES Checklist. (DOCX)

## References

[1] Bildirici M, Sunal S, Aykac Alp E, Orcan M. Determinants of human capital theory, growth and brain drain: An econometric analysis for 77 countries. Appl Econometr Int Dev. 2005;5(2):2.

[2] Turin TC, Chowdhury N, Haque S, Rumana N, Rahman N, Lasker MAA. Meaningful and deep community engagement efforts for pragmatic research and beyond: engaging with an immigrant/racialised community on equitable access to care. BMJ Glob Health. 2021;6(8):e006370. doi: 10.1136/bmjgh-2021-006370 34426405 PMC8383879

[3] Immigration, Refugees and Citizenship Canada Departmental Plan 2018–2019. Ottawa: Immigration, Refugees and Citizenship Canada. 2018. Available from: https://www.canada.ca/en/immigration-refugees-citizenship/corporate/publications-manuals/departmental-plan-2018-2019/departmental-plan.html

[4] Al-Haddad M. International medical graduates: defining the term and using it consistently. BMJ Glob Health. 2024;9(8):e015678. doi: 10.1136/bmjgh-2024-015678 39153749 PMC11331860

[5] Chowdhury N, Ekpekurede M, Lake D, Chowdhury TT. The alternative career pathways for international medical graduates in health and wellness sector. International Medical Graduates in the United States: A Complete Guide to Challenges and Solutions. Springer; 2021. pp. 293–325.

[6] Azemi A, Parra-Herran C. International medical graduates representation in pathology academic workforce, departmental leadership and society leadership. Acad Pathol. 2025;12(1):100158. doi: 10.1016/j.acpath.2024.100158 39877832 PMC11773459

[7] The state of medical education and practice in the UK. Workforce report. 2025.

[8] OECD. Health at a Glance 2023: OECD Indicators. OECD Publishing; 2023. doi: 10.1787/7a7afb35-en

[9] Turin TC, Chowdhury N, Lake D. Alternative careers toward job market integration: barriers faced by International Medical Graduates in Canada. Int J Environ Res Public Health. 2023;20(3):2311. doi: 10.3390/ijerph20032311 36767681 PMC9915349

[10] A profile of physicians in Canada. Canadian Institute for Health Information. Available from: https://www.cihi.ca/en/a-profile-of-physicians-in-canada

[11] Statistics Canada. Internationally educated health care professionals in Canada: Sociodemographic characteristics and occupational distribution. 2023 [cited 26 Aug 2025]. doi: 10.25318/36280001202300800004-ENG

[12] Frank K, Park J, Cyr P, Weston S, Hou F. Internationally educated health care professionals in Canada: sociodemographic characteristics and occupational distribution. 2023.

[13] Wang Y, Das RLV, Lapa T, Marosan P, Pawliuk R, Chable HD, et al. Career development of international medical graduates in Canada: status of the unmatched. Humanit Soc Sci Commun. 2023;10(1):38. doi: 10.1057/s41599-023-01534-z 36741982 PMC9885407

[14] Leiva Tobelem LF, Ynoe de Moraes F, Santos FNC. Expanding healthcare capacity in Canada: the potential of internationally trained physicians. Lancet Reg Health Am. 2025;46:101095. doi: 10.1016/j.lana.2025.101095 40290133 PMC12032375

[15] Canadian Resident Matching Service. 2022 CaRMS Forum. Canadian Resident Matching Service (CaRMS); 2022.

[16] MacFarlane MM. When a Canadian is not a Canadian: marginalization of IMGs in the CaRMS match. Can Med Educ J. 2021;12(4):132–40. doi: 10.36834/cmej.71790 34567315 PMC8463232

[17] Turin TC, Chowdhury N, Ekpekurede M, Lake D, Lasker MAA, O’Brien M, et al. Professional integration of immigrant medical professionals through alternative career pathways: an Internet scan to synthesize the current landscape. Hum Resour Health. 2021;19(1):51. doi: 10.1186/s12960-021-00599-8 33865402 PMC8052743

[18] Turin TC, Chowdhury N, Ekpekurede M, Lake D, Lasker M, O’Brien M, et al. Alternative career pathways for international medical graduates towards job market integration: a literature review. Int J Med Educ. 2021;12:45–63. doi: 10.5116/ijme.606a.e83d 33839694 PMC8415394

[19] Turin TC, Chowdhury N, Lake D. Lost in transition: the need for a strategic approach to facilitate job market integration of internationally educated physicians through alternative careers. Int J Environ Res Public Health. 2022;19(6):3503. doi: 10.3390/ijerph19063503 35329190 PMC8954027

[20] Bourgeault IL, Baumann A. Ethical recruitment and integration of internationally educated health professionals in Canada. 13th International Health Workforce Collaborative. 2011.

[21] Turin TC, Chowdhury N, Lake D, Chowdhury MZ. Labor market integration of high-skilled immigrants in Canada: Employment patterns of international medical graduates in alternative jobs. MDPI; 2022. pp. 1705. 10.3390/healthcare10091705 PMC9498913 36141317

[22] Turin TC, Chowdhury N, Haque S, Rumana N, Rahman N, Lasker MAA. Involving im/migrant community members for knowledge co-creation: the greater the desired involvement, the greater the need for capacity building. BMJ Glob Health. 2021;6(12):e007602. doi: 10.1136/bmjgh-2021-007602 34969687 PMC8718487

[23] Qualtrics: survey research suite. Available from: https://www.qualtrics.com

[24] Stratton SJ. Population research: convenience sampling strategies. Prehosp Disaster Med. 2021;36(4):373–4. doi: 10.1017/S1049023X21000649 34284835

[25] McHugh ML. The chi-square test of independence. Biochem Med (Zagreb). 2013;23(2):143–9. doi: 10.11613/bm.2013.018 23894860 PMC3900058

[26] Warner P. Testing association with Fisher’s Exact test. J Fam Plann Reprod Health Care. 2013;39(4):281–4. doi: 10.1136/jfprhc-2013-100747 24062499

[27] Abedin T, Chowdhury Z, Afzal AR, Yeasmin F, Turin TC. Application of binary logistic regression in clinical research. JNHFB. 2016;5:8–11.

[28] Chowdhury MZI, Turin TC. Variable selection strategies and its importance in clinical prediction modelling. Fam Med Community Health. 2020;8(1):e000262. doi: 10.1136/fmch-2019-000262 32148735 PMC7032893

[29] Eysenbach G. Improving the quality of Web surveys: the Checklist for Reporting Results of Internet E-Surveys (CHERRIES). J Med Internet Res. 2004;6(3):e34. doi: 10.2196/jmir.6.3.e34 15471760 PMC1550605

[30] Shommu N, Choudhury SR, Turin TC. Knowledge translation in health care: bridging the gap between “knowledge generation” and “knowledge implementation”. JNHFB. 2017;6:2–4.

[31] Guidelines for effective knowledge mobilization. Social Sciences and Humanities Research Council, Canada. 2019. Available from: http://www.sshrc-crsh.gc.ca/funding-financement/policies-politiques/knowledge_mobilisation-mobilisation_des_connaissances-eng.aspx

[32] Guide to knowledge translation planning at CIHR: integrated and end-of-grant approaches. Canadian Institutes of Health Research. 2015. Available from: http://www.cihr-irsc.gc.ca/e/45321.html

[33] Turin TC, Chowdhury N, Rumana N, Lasker MAA, Qasqas M. Partnering with organisations beyond academia through strategic collaboration for research and mobilisation in immigrant/ethnic-minority communities. BMJ Glob Health. 2022;7(3):e008201. doi: 10.1136/bmjgh-2021-008201 35332054 PMC8948381

[34] Neiterman E, Bourgeault IL, Covell CL. What Do We Know and Not Know about the Professional Integration of International Medical Graduates (IMGs) in Canada? Healthc Policy. 2017;12(4):18–32. doi: 10.12927/hcpol.2017.25101 28617235 PMC5473472

[35] Sikdar S, Chowdhury N, Lake D, Turin TC. Alternative career pathway decision-support job database for international medical graduates in Canada. BMC Res Notes. 2022;15(1):336. doi: 10.1186/s13104-022-06232-8 36309723 PMC9617379

