# Supplementary material for: Who considers alternative employment pathways among International Medical Graduates in Canada?
Source: PLoS One. 2026 Aug 13;21(8):e0355462. doi: 10.1371/journal.pone.0355462 (PMC13472413; doi:10.1371/journal.pone.0355462)
Supplement: Supplementary file 1 [file pone.0355462.s001.docx]

Appendix: Survey Questionnaire

**Opportunities and challenges for International Medical Graduates (IMGs) in contributing to community health and wellness**

1. **Your age?**
2. **Your sex?**
3. **Country of origin?**
4. **In what year did you arrive in Canada?**
5. **Which province/territories did you land upon arrival?**
6. **Which province/territories are you currently living in?**
7. **What is your immigration status?**
8. **Which country did you obtain your medical graduation from?**
9. **In what year did you complete your medical graduation?**
10. **What was your highest level of practice before you came to Canada?**
11. **What was your specialty before you came to Canada?**
12. **What post-graduate degrees/training have you obtained after graduation? (e.g. MRCP, FRCS, MD, MS, PGD etc. )**
13. **Which countries did you work as a physician/healthcare professional before moving to Canada?**
14. **What is your current work position?**
15. **What is your current area of work?**
16. **Which of the following range best describes your current income (yearly)?**
17. **What is your current career goal?**
18. **What steps of licensure process have you completed already? Please check all the steps you completed and write the number of times you have attempted each.**
19. **How much time have you devoted to the pursuit of medical licensure?**
20. **How much money have you spent on the pursuit of medical licensure? (range)**
21. **How confident are you in attaining medical licensure?**
22. **If you are employed in an alternative career path, are you still pursuing medical licensure?**
23. **To what extent do you agree that the following issues are barriers to your pursuit of medical licensure?**

|  | *Strongly agree* | *Somewhat agree* | *Neither agree nor disagree* | *Somewhat disagree* | *Strongly disagree* | *Strongly agree* |
| --- | --- | --- | --- | --- | --- | --- |
| ***Financial strain*** |  |  |  |  |  |  |
| ***Family responsibilities*** |  |  |  |  |  |  |
| ***Lack of coaching and guidance*** |  |  |  |  |  |  |
| ***Lack of information*** |  |  |  |  |  |  |
| ***Lack of 'Canadian experience' (e.g., observership, volunteering etc.)*** |  |  |  |  |  |  |
| ***Failure to acculturate*** |  |  |  |  |  |  |
| ***Time out of practice*** |  |  |  |  |  |  |
| ***Failure to obtain required score in English Language Proficiency test*** |  |  |  |  |  |  |

24. **Are you required to write the *IELTS* exam to pursue medical licensure?**
25. **To what extent is attaining the required *IELTS score* a barrier to your pursuit of medical licensure?**
26. **How many times have you attempted to obtain required IELTS score?**
27. **Which section (s) of IELTS exam have you been failing to obtain the required score? (if applicable)**
28. **If you have faced any other barriers to obtaining the medical licensure, please describe below**
29. **Are you considering for an alternative career path?**
30. **What type of alternative career path are you considering? (If yes to previous question)**
31. **Have you obtained any courses/degrees/training related to an alternative career or are you currently enrolled in any (*regulated/unregulated/non-health*)?**
32. **How much time have you devoted to pursuing *an alternative career* (*regulated/unregulated/non-health*)?**
33. **How confident are you to attaining an alternative career? (If applicable)**
34. **How much money have you spent pursuing a *regulated* alternative career path? (i.e., *requires licensure*; e.g., nursing, laboratory technician, EMS technician etc.)?**
35. **How much money have you spent pursuing an *unregulated* alternative career options? (i.e., requires *no licensure*; e.g., health educator, health administrative officer, researcher, health policy analyst etc.)?**
36. **How much money have you spent pursuing a *non-health related* alternative career options? (e.g., engineering, business, life sciences etc.)?**
37. **To what extent do you agree that *the following issues are* barriers to your pursuit of alternative career path?**

|  | *Strongly agree* | *Somewhat agree* | Neither agree nor disagree | *Somewhat disagree* | *Strongly disagree* |
| --- | --- | --- | --- | --- | --- |
| ***Financial strain*** |  |  |  |  |  |
| ***Family responsibilities*** |  |  |  |  |  |
| ***Lack of guidance and direction*** |  |  |  |  |  |
| ***Lack of information*** |  |  |  |  |  |
| ***Lack of confidence*** |  |  |  |  |  |
| ***Peer pressure (i.e., friends and family influence/persuasion to become a practising physician)*** |  |  |  |  |  |
| ***Lack of required skills for alternative career*** |  |  |  |  |  |
| ***Lack of bridging/skills-building programs for alternative career*** |  |  |  |  |  |
| ***Lack of relevant experience from previous work (e.g., research experience, laboratory experience etc.)*** |  |  |  |  |  |
| ***Lack of relevant 'Canadian experience' (e.g., volunteering opportunity for research or in laboratory settings etc.)*** |  |  |  |  |  |
| ***Failure to acculturate (i.e., acquiring and adapting to Canadian culture and norms)*** |  |  |  |  |  |
| ***Lack of sufficient communication/linguistic skills*** |  |  |  |  |  |

38. **If you have faced any other barriers to obtaining your preferred *alternative career path*, please describe below.**
39. **How involved are you to improve your community health and well-being?**
40. **How often are you called upon by your community members to provide health related information, advice or guidance?**
41. **Have you conducted any health-related Knowledge Mobilisation (KM) activities i.e. workshops, newsletter, symposium, posters etc. in the communities to your capacity?**
42. **What Knowledge Mobilisation (KM) activities have you conducted?**
43. **How engaged are you in conducting these types of activities as listed above in the communities?**
44. **What are the barrier (s) that prevented you or that you had to overcome to conduct health-related Knowledge Mobilisation activities in the communities?**
45. **How interested are you in conducting Knowledge Mobilisation (KM) activities about the health and wellness in the communities?**
46. **How confident are you in your ability to conduct Knowledge Mobilisation (KM) activities?**
47. **Do you have any formal training to conduct any Knowledge Mobilisation activities?**
48. **We will arrange a raffle draw within the participants of this survey and offer $10 e-transfers to 50 winners. If you are interested to participate in the prize draw, please provide your contact information on the added page (FOR RAFFLE DRAW PARTICIPANTS). So that if you win we can contact and deliver the e-transfer to you. (Optional)**
